# BaTiO_3_–(Na_0.5_Bi_0.5_)TiO_3_ Ceramic Materials Prepared via Multiple Design Strategies with Improved Energy Storage

**DOI:** 10.3390/nano15221724

**Published:** 2025-11-15

**Authors:** Jianming Deng, Jingjing Guo, Ting Wang, Jingxiang Zhang, Xu Wu, Xuefeng Zhang, Surya Veerendra Prabhakar Vattikuti, Qing Ma, Pitcheri Rosaiah, Qingfeng Zhang

**Affiliations:** 1 Guangdong Provincial Key Laboratory of Electronic Functional Materials and Devices, Huizhou University, Huizhou 516001, China; 2 Shenzhen Key Laboratory of Advanced Materials, School of Materials Science and Engineering, Harbin Institute of Technology, Shenzhen 518055, China; 3 School of Mechanical Engineering, College of Engineering, Yeungnam University, Gyeongsan 38541, Republic of Korea; 4 Department of Physics, Saveetha School of Engineering, Saveetha Institute of Medical and Technical Sciences (SIMATS), Chennai 602105, India; dr.mosesrosaiah@gmail.com; 5 Hubei Key Laboratory of Micro-Nanoelectronic Materials and Devices, Hubei University, Wuhan 430062, China; zhangqingfeng@hubu.edu.cn

**Keywords:** barium titanate, lead-free, ceramic capacitors, Ca_0.7_La_0.2_(Mg_1/3_Nb_2/3_)O_3_, energy storage

## Abstract

The investigation of environmentally friendly, Pb-free ceramic dielectric materials with excellent energy storage capability represents a fundamental yet challenging research direction for the development of next-generation high-power capacitors. In this study, linear dielectric Ca_0.7_La_0.2_(Mg_1/3_Nb_2/3_)O_3_ was added into [0.65BaTiO_3_–0.35(Na_0.5_Bi_0.5_)TiO_3_] to form a solid solution. The introduction of Ca_0.7_La_0.2_(Mg_1/3_Nb_2/3_)O_3_ modified the crystal structure, enhanced insulation performance and breakdown strength, and reduced hysteresis loss. These improvements collectively contributed to higher energy storage density and efficiency (*η*). The ceramic pellet with the optimal 10 mol% Ca_0.7_La_0.2_(Mg_1/3_Nb_2/3_)O_3_ demonstrated a higher retrievable energy density (~3.40 J cm^−3^) and efficiency (~81%) at a breakdown strength of 340 kV cm^−1^ compared to BaTiO_3_-based ferroelectric ceramics. The sample also exhibited good stability across a temperature range of 30–90 °C and a frequency range of 0.5–300 Hz. Thus, the as-prepared ceramics sample exhibited significant potential for pulsed power device applications.

## 1. Introduction

The rapid rise in global energy consumption over recent decades has driven the demand for renewable, efficient, and environmentally friendly energy storage devices [1,2,3,4]. Dielectric capacitors, as essential components of modern electronic systems, can store energy electrostatically and are widely employed for energy storage due to their ability to rapidly and reliably store and release energy [5,6]. When an electric field is applied, the positive and negative charges inside the material’s atomic structure are slightly pulled apart. Energy is stored within this induced polarization. After the electric field is removed, the material releases this stored energy as it discharges. However, their relatively low energy storage density remains a significant limitation for pulsed power applications. Consequently, there is an urgent need for environmentally friendly (lead-free) energy storage materials that have both a high recoverable energy storage density (*W*_rec_) and high energy storage efficiency (*ƞ*) [7,8]. Generally, the total energy storage density (*W*), *W*_rec_, and *ƞ* are expressed as Equations (1)–(3) [9]:

(1) $$W=\int_{0}^{P_{\max}}EdP$$

(2) $$W_{\mathrm{rec}}=\int_{P_{r}}^{P_{\max}}EdP$$

(3) $$\eta =\frac{W_{\mathrm{rec}}}{W}$$

where *P*_max_, *P*_r,_ and *P* indicate the maximum, remnant, and normal polarization, respectively; *E* denotes the applied electric field. *W*_rec_ can be improved by simultaneously achieving Δ*P* (*P*_max_ − *P*_r_) and a high *E*.

Energy storage ceramic materials with dielectric properties are generally classified into four categories: ferroelectric (FEc), linear dielectric, antiferroelectric (AFEc), and relaxor ferroelectric (RFc) ceramics [10,11]. RFc and AFEc ceramics are regarded as excellent energy storage materials owing to their high *P*_max_. However, many AFEc systems contain expensive noble metals such as silver or environmentally hazardous elements like lead [12,13,14]. RFc materials are particularly attractive because they contain polar nano-regions (PNRs). These PNRs enable rapid domain switching under an applied *E*, resulting in a small *P*_r_. This, combined with their high *P*_max_ values, allows them to simultaneously achieve excellent *W*_rec_ and *η*. Consequently, lead-free RFc materials are gaining significant attention for both environmental and economic reasons.

Barium titanate (BaTiO_3_, BT), the first discovered perovskite-type piezoelectric material, has been extensively utilized in commercial multilayer ceramic capacitors [15]. However, the low intrinsic breakdown electric field (*E*_b_) and high coercivity field (*E*_c_) of these devices limit a desirable *W* value. The fast response of polar nanoregions (PNRs) reduces *E*_c_ in the presence of electric fields, which substantially improves the efficiency (*η*) and energy density (*W*) [16,17,18,19]. Lv et al. [20] added 40 mol% of strontium bismuth titanate (Sr_0.7_Bi_0.2_TiO_3_) to a 0.6 [0.94 sodium bismuth titanate (Na_0.5_Bi_0.5_TiO_3_, NBT)–0.06 BT] system. The macroscopic domain was disrupted and *P*_r_ and *E*_c_ were reduced, which enhanced *W* and *η*. In addition, Yuan et al. [21] enhanced the threshold field and decreased *E*_c_ for introducing a long-range order in a BT–bismuth magnesium zirconate [Bi(Mg_0.5_Zr_0.5_)O_3_] system by forming PNRs. While various modification strategies exist, a long-term challenge remains in further improving the energy storage capacity of these ceramics [16].

Sodium bismuth titanate (Na_0.5_Bi_0.5_TiO_3_, NBT) is a lead-free ceramic material for energy storage. Its high *P*_max_ (>40 μC cm^−2^) arises from the hybridization between the O^2−^ 2*p* orbitals and the Bi^3+^ 6*s*^2^ lone pair electrons [22]. Incorporating NBT into BaTiO_3_ effectively enhances polarization and stabilizes dielectric performance. This additional O 2*p*-Bi 6*s* orbital hybridization compensates for the polarization deficiency in BT [23]. As a result, 0.65BT-0.35NBT-based ceramics have been extensively researched for their excellent *W*. High energy storage performance has been reported for (1–*x*)(0.65BT–0.35NBT)– *x*Bi(Mg_2/3_Nb_1/3_)O_3_ materials with *η* of 90.3% and *W*_rec_ of 1.6 J cm^−3^ [24]. Similarly, Dai et al. [25] et al. achieved *η* of 90.18% and *W*_rec_ of 2.02 J cm^−3^ at 206 kV cm^−1^ by adding Sr(Sc_0.5_Nb_0.5_)O_3_ into 0.65BT-0.35NBT ceramics.

Herein, 0.65BT–0.35NBT was selected as a basic component, while Ca_0.7_La_0.2_(Mg_1/3_Nb_2/3_)O_3_ (CLMN), a linear dielectric material, was used for doping 0.65BT–0.35NBT. This is a rational, multifunctional design strategy. The effect of CLMN on the energy storage performance of 0.65BT–0.35NBT was investigated. Calcium titanate (CaTiO_3_) is a linear dielectric material with a high *E*_b_ and negligible *P*_r_. Incorporating CaTiO_3_ into ferroelectric matrices to form solid solutions can enhance *W* [26,27]. The addition of the rare earth element lanthanum (La) is known to refine grains [28], while the presence of magnesium oxide (MgO) and niobium pentoxide (Nb_2_O_5_) with their wide bandgaps (*E*_g_, 7.8 eV and 3.4 eV, respectively) improves the material’s insulation performance. These improved relaxation behavior, and reduced *P*_r_ in CLMN-doped 0.65BT–0.35NBT, optimizing its energy storage properties. Introducing 10 mol% of CLMN into the ceramic significantly enhanced its energy storage performance, with an *E*_b_ of 340 kV cm^−1^, *η* of ~81%, and a *W*_rec_ of ~3.40 J cm^−3^. The resulting ceramic also demonstrated good stability over a temperature range of 30–90 °C and a frequency range of 0.5–300 Hz. This approach provides an effective strategy to optimize the energy storage capability of BT-based ceramics, making them suitable for advanced power systems.

## 2. Experimental Procedure

(1–*x*)[0.65BaTiO_3_–0.35(Na_0.5_Bi_0.5_)TiO_3_]–*x*Ca_0.7_La_0.2_(Mg_1/3_Nb_2/3_)O_3_ (abbreviated as *x*CLMN, *x* = 0.08, 0.10, 0.12, 0.14 and 0.16) lead-free ceramic samples were synthesized using high-purity raw materials: bismuth oxide (Bi_2_O_3_, AR 99%), sodium carbonate (Na_2_CO_3_, AR 99.8%), barium carbonate (BaCO_3_, AR 99%), calcium carbonate (CaCO_3_, AR 99%), lanthanum oxide (La_2_O_3_, metal basis 99.9%), magnesium oxide (MgO, AR 98%), niobium oxide (Nb_2_O_5_, Metals basis 99.9%) and titanium oxide (TiO_2_, AR 99%) using a standard solid-state method. The chemicals were procured from Alladin Scientific, Shanghai, China. The powders and ceramic pellets were prepared according to the procedure described in our previous work [29]. The powders were weighted according to the stoichiometric ratio and ball milled in ethanol for 24 h. After drying, the mixtures were calcined at 900 °C for 4 h in air. The calcined powders were milled again and pressed into disks with 12 mm in diameter and 1.5 mm in thickness using 6 wt% PVA aqueous solution as binder. The green pellets were heated to 600 °C for 6 h to burn out the binder. The pellets were sintered in a covered alumina crucible at 1050–1170 °C for 3 h to obtain high density.

The crystal structures were characterized using a Renishaw Raman spectrometer and X-ray diffraction (XRD) on SmartLab (Rigaku, Tokyo, Japan) instruments under the following conditions: (i) step size, 0.02°; (ii) 2θ range, 20–80°; and (iii) scanning speed, 2° min^−1^. Raman spectroscopy (Renishaw InVia Reflex) with a radiation of Ar^+^ laser (λ = 514.5 nm) was carried out to investigate the structural properties of ceramics at room temperature. The thermally etched surface morphology of the ceramic pellets was examined via scanning electron microscopy (SEM; VEGA3/XUM, TESCAN, Brno, Czechia), and elemental composition was analyzed using energy-dispersive X-ray spectroscopy (EDS) during SEM analysis. Grain size distribution of the samples was determined using Nano Measurer software 1.2. The ceramic samples were manually ground and polished to a thickness of 0.5 mm for electrode fabrication. Silver electrodes were applied and subsequently heated at 550 °C for 30 min. Impedance spectra and dielectric properties were measured using an impedance analyzer (Keysight E4990A, Santa Rosa, CA, USA) at a heating rate of 3 °C min^−1^ over a temperature range of 30–200 °C and at various frequencies. The ferroelectric properties were measured using unipolar triangular waveform at 1 Hz and under varying electric fields using a Premier II ferroelectric tester (Radiant Technologies Inc., New Mexico, NW, USA). The green pellets were polished to a appropriately 0.15 mm thickness prior to testing. Au electrodes with a 3.14 mm^2^ area were sputtered.

## 3. Results and Discussion

**Figure 1:**
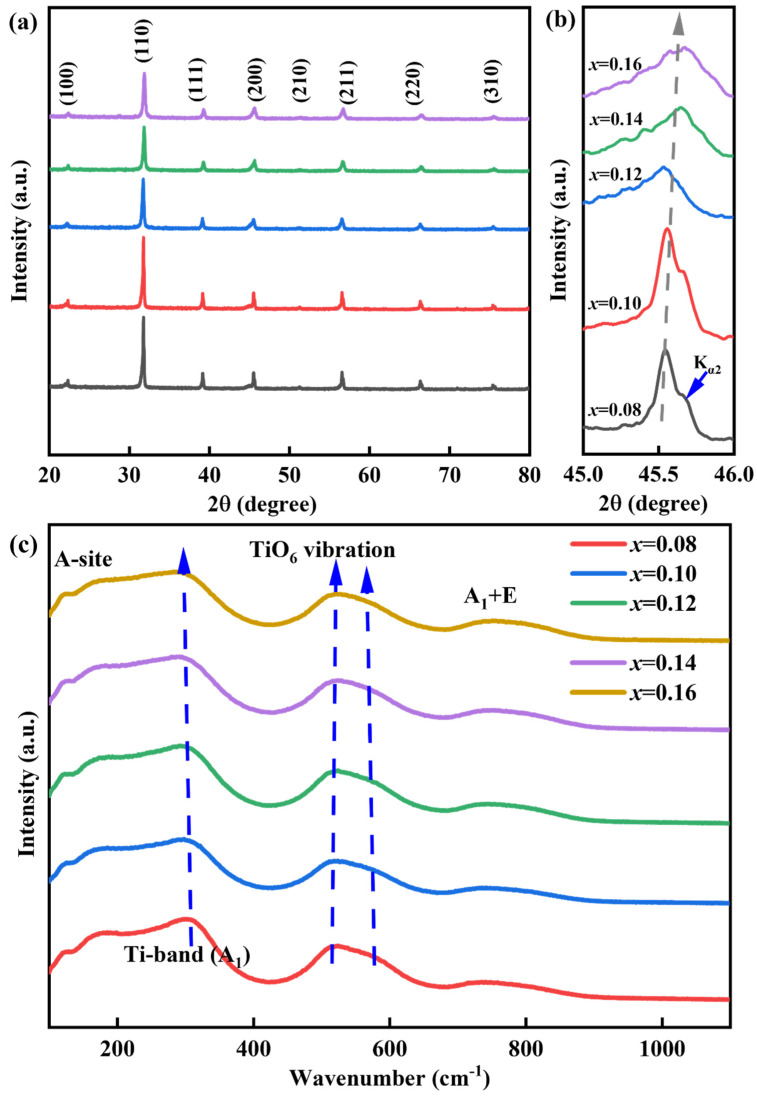
(**a**) Room-temperature XRD patterns of as-prepared ceramics; (**b**) the corresponding magnification around 2θ ∼ 45°; (**c**) Raman spectra of the *x*CLMN ceramics.

The phase structure of *x*CLMN samples was analyzed via XRD, and the results are presented in Figure 1. The XRD patterns of the samples exhibited a pure perovskite structure. The absence of secondary phases indicated that Ca, La, Mg, and Nb ions were successfully incorporated into the BT-NBT lattice, forming a solid solution. With increasing CLMN doping concentration, the symmetry of the (200) diffraction peak at the 2θ value of 45° increased (Figure 1b), suggesting a transition from the tetragonal phase (*T*, *P4mm*) to the pseudo-cubic phase (*PC*, *Pm-3m*) [30]. The presence of the K_a2_ reflection, often mistaken for non-cubic distortion, is a key observation [31]. This reflection is typically difficult to resolve in tetragonal or rhombohedral symmetries. The distinct appearance of the (200) peak at low diffraction angles (<50°) indicates that non-cubic distortions were negligible within the detection limits of the X-ray apparatus. Further, with increasing CLMN content, the peaks shifted toward higher 2θ values, suggesting a reduction in the lattice constant and unit cell volume. This effect likely arises because the ionic radii of the dopants (Ca^2+^, Mg^2+^, Nb^5+^, and La^3+^) are smaller than those of the original A-site and B-site ions [23,30].

The structure and phase evolution of *x*CLMN samples were analyzed via Raman spectroscopy (Figure 1c). BT-based ferroelectric ceramics exhibit four main vibrational modes [30,31,32,33], which were also observed in our sample spectra: (i) A-site cations vibrations (<200 cm^−1^), (ii) B–O bond vibrations (200–400 cm^−1^), (iii) BO_6_ octahedra vibrations (400–650 cm^−1^), and (iv) A_1_ + E (>700 cm^−1^) overlapping bands. The Raman bands at ~179 and 120 cm^−1^ correspond to A–O vibrations, confirming the presence of Na^+^, Ba^2+^, Bi^3+^, La^3+^, and Ca^2+^. Furthermore, the bending vibrational mode (A_1_) of the Ti–O bond at ~300 cm^−1^ shifted to a lower wavenumber. With increasing CLMN concentration, the Raman peaks near 300 cm^−1^ became broader and more diffuse. Multiple cations occupy the A-site causing a mismatch in the valency, which induces a high local disorder and reduced unit cell polarity [34,35]. It can be induced that CLMN doping progressively replaces the Ti^4+^ ions, which reduces the material’s *P* [30]. The creation of local nanoregions is proved by the appearance of the Raman peaks at ~750 cm^−1^ [36]. Consequently, adding CLMN breaks down the long-range ferroelectric order, leading to the formation of PNRs [37]. This makes the material exhibit a high *P*_max_ and a nearly negligible *P*_r_ during charging and discharging. The transformation of the Raman peaks from active modes to complex/diffused modes is similar to those of the XRD peaks. This indicates a phase transition from the tetragonal (ferroelectric) state to a pseudo-cubic (relaxor ferroelectric) state.

**Figure 2:**
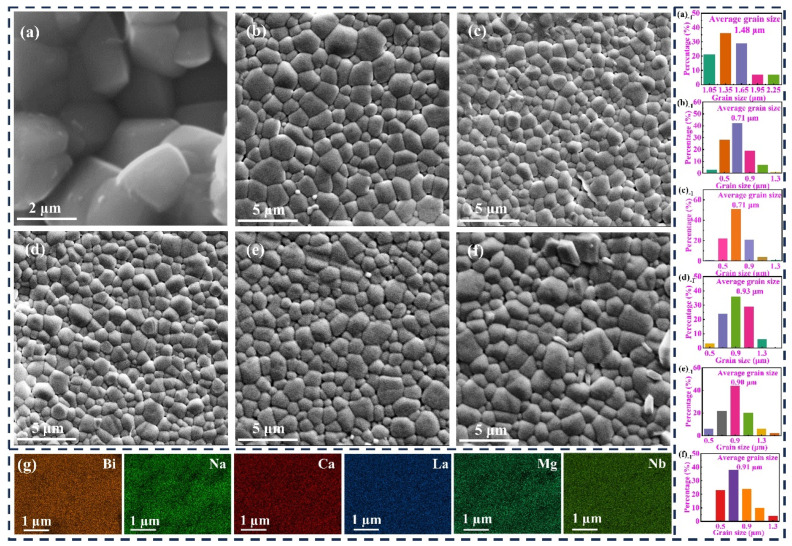
(**a**–**f**) SEM micrographs and grain size for ceramics sintered at optimal sintering temperature; (**g**) SEM-EDS images of 0.1CLMN ceramic.

The thermally etched surface morphology of the ceramic samples was analyzed using SEM (Figure 2). The *x*CLMN ceramics exhibited a dense microstructure with well-defined grain boundaries and a few pores. Grain-size distributions were calculated using analytical software, and variations in grain size among the as-sintered samples were assessed (Figure 2a_−1_–f_−1_). The grain sizes of CLMN-doped ceramics decreased from 1.48 μm to 0.9 μm. This reduction is crucial for achieving a high *E*_b_ and is closely related to grain-boundary migration and the sintering temperature. Moreover, the ceramic grain size was highly sensitive to the CLMN content. EDS mapping of 0.10CLMN (Figure 2g) revealed compositional homogeneity, with all elements uniformly distributed across the observed region, showing no signs of segregation or aggregation.

**Figure 3:**
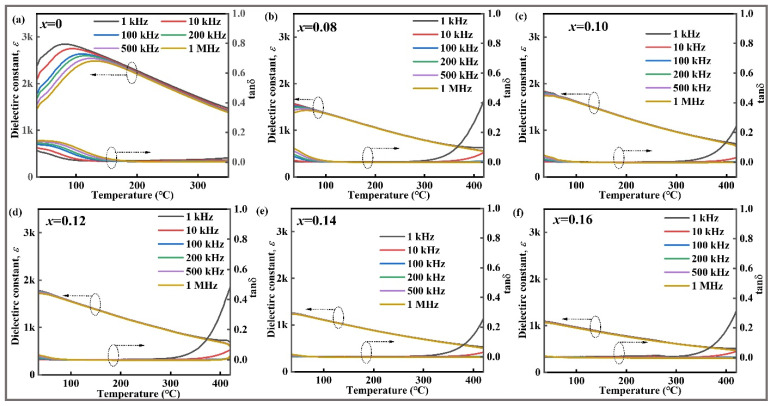
Temperature-dependent dielectric constant and tanδ for all ceramics at different frequency: (**a**) *x* = 0; (**b**) *x* = 0.08; (**c**) *x* = 0.10; (**d**) *x* = 0.12; (**e**) *x* = 0.14; (**f**) *x* = 0.16.

The behavior of different polar components in a material under an external field with a low alternating current is indicated by the dielectric properties of the material. Based on this phenomenon, the relaxation properties and phase transition of ferroelectrics were investigated. The loss tangent (tan δ) and dielectric constant (*ε*) of *x*CLMN were measured as a function of temperature at various frequencies, and the results are shown in Figure 3. For ferroelectric materials, the sharp peaks representing dielectric components are independent of frequencies. However, an increase in the CLMN concentration gradually alters the sharp high *ε*-temperature peak (*T*_m_) to a diffuse and broad peak. This phenomenon is attributed to frequency dispersion, highlighting the existence of pronounced relaxor characteristics [38]. Furthermore, *T*_m_ and the maximum dielectric constant (*ε*_m_) decreased with increasing *x* amount. The reduction in *ε*_m_ is likely due to changes in the lattice constant and A-site ionic radii [39]. The presence of different cations at the A-site compresses the oxygen octahedra and reduces the mobility of B-site cations. Therefore, *ε* and *P* are lowered. The domain structure might also be involved. An increase in the *x* content diminishes the phase proportion of the *T* phase whereas that of the *PC* phase increases, forming a relaxor ferroelectric phase. The presence of these nanodomain structures might decrease *ε*_m_ [39]. Notably, *T*_m_ shifts to room temperature for samples with *x* ≥ 0.10.

**Figure 4:**
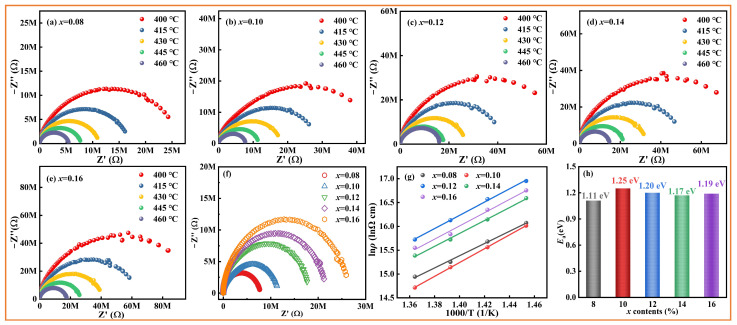
Impedance spectra of *x*CLMN ceramics measured at various temperatures: (**a**) *x* = 0.08; (**b**) *x* = 0.10; (**c**) *x* = 0.12; (**d**) *x* = 0.14; (**e**) *x* = 0.16; (**f**) composition-dependent impedance spectra of as-prepared ceramics at 445 °C; (**g**) Arrhenius-type plots of resistivity vs. 1000/T of as-prepared ceramics; (**h**) variation of *E*_a_ value with doping content).

The conductivity, dielectric behavior, and relaxation-type characteristics of *x*CLMN ceramic materials were investigated via impedance spectroscopy. The impedance spectra of the samples from 400 °C to 460 °C in the range of 20–10^6^ Hz are presented in Figure 4. The samples exhibited a semicircular feature in the examined temperature range, likely due to grain boundaries [40]. The impedance data were fitted using a single parallel resistor–capacitor equivalent circuit to extract reliable resistance values for the grain boundary phases. Grain boundary conductivity displayed an evident decrease in impedance at high temperatures, indicating a thermally activated conduction behavior [40]. Plotting the composition-dependent impedance spectra at 445 °C [Figure 4f] reveals a dramatic increase in the semicircle radius as the CLMN concentration rises. This clearly indicates a corresponding increase in insulating resistance. In addition, the Arrhenius law was used to calculate the required activation energy (*E*_a_) for electrical conductivity [Equations (4) and (5)] [17,40]:

(4) $$\sigma =\sigma_{0}\exp\left(-\frac{E_{a}}{k_{B}T}\right)$$

(5) $$\sigma =\frac{l}{RS}$$

where *k*_B_ denotes the Boltzmann constant, *σ*_0_ denotes a pre-exponential factor, and *σ* denotes electrical conductivity. *S* denotes the cross-sectional sample area, and *R* represents the real-part impedance (Z′) extrapolated intercept. T and l denote the measured temperature and sample thickness, respectively. Figure 4g shows the plot of ln ρ against 1000/T; The solid line is fit using Equation (4). The *E*_a_ of the *x*CLMN samples was ascertained by fitting the conductivity at high temperatures linearly (1.10–1.30 eV) [Figure 4h]. The B- and A-site values of cation transport are 14 and 4 eV, respectively, and the resulting *E*_a_ values of the samples are 1.11–1.25 eV, which is similar to *E*_a_ required for the migration of the oxygen vacancy (*E*_a_ < 2 eV) [41]. Therefore, Therefore, oxygen vacancy dictates the conductivity of the samples at high temperatures, which is in agreement with reported BT-based ceramics [19,41,42]. It is noted that when the CLMN content rises, the *E*_a_ of the 0.10CLMN ceramics peaks at 1.25 eV.

**Figure 5:**
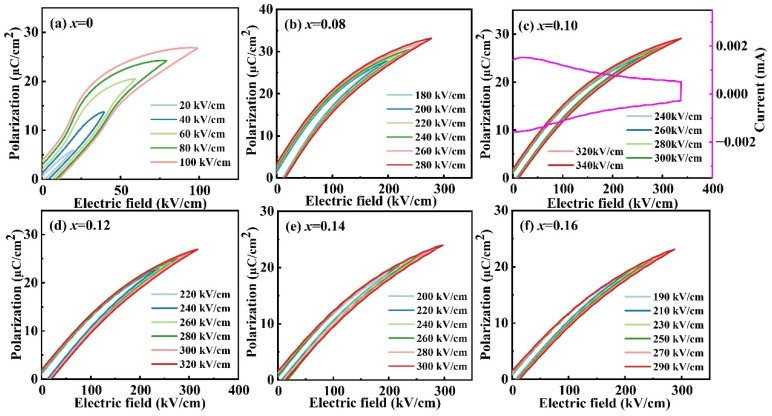
Unipolar *P*-*E* loops under different applied electric fields for xCLMN ceramics: (**a**) x = 0; (**b**) x = 0.08; (**c**) x = 0.10; (**d**) x = 0.12; (**e**) x = 0.14; (**f**) x = 0.16. (the pink line in (**c**): the current-electric field curve).

The ferroelectric performance of *x*CLMN ceramics (Figure 5) is exhibited by their ferroelectric hysteresis *P*–electric field (*E*) loops under the applied electric field with a maximum value. With increasing CLMN content, the *P-E* loop gradually shifted from one with a large *P*_r_ and high *E*_c_ to a more linear shape, indicating that the long-range ferroelectric order was gradually disrupted, leading to the formation of nanodomains or PNRs [23]. The PNRs or nanodomains are dependent on the applied electric field. Under an external electric field, they exhibit a long-range ferroelectric state, while in the absence of the field, they revert to the original ergodic state. This behavior is consistent with the dielectric measurement results shown in Figure 3. A lower *P*_r_ and moderate *P*_max_ are obtained in 0.10CLMN ceramics, which has a peak value of *E*_b_.

**Figure 6:**
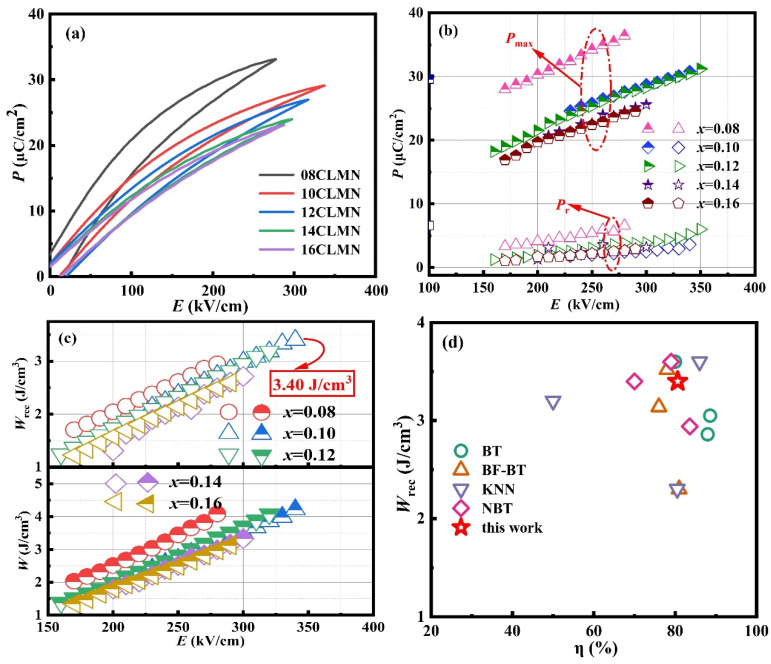
(**a**) Unipolar *P-E* loops prior to their breakdown electric fields; (**b**) *P*_max_, *P*_r_ and (**c**) *W*, *W*_rec_ change with *E* for *x*CLMN ceramics; (**d**) A comparison of *W*_rec_ and *η* in this study and reported in other lead-free bulk ceramics.

Figure 6a revealed the *P*–*E* loops of *x*CLMN ceramic materials determined at room temperature under a critical electric field. With increasing the CLMN content, the ferroelectric hysteresis loop of the ceramic samples narrowed, accompanied by a gradual decrease in the polarization (*P*) value. The observed phenomenon was attributed to the presence of the *PC* phase, which is consistent with the findings derived from the dielectric and XRD analyses. The energy storage properties of *x*CLMN ceramic materials are presented in Figure 6b,c, showing the critical values *P*_max_, *P*_r_, *W*_rec,_ and *W* of *P*–*E* as a function of *E*. *P*_r_ demonstrated minimal variation with an increase in *x*, revealing the characteristic relaxor behavior. Upon removing the electric field, a transition occurred from a more coherent long-range ferroelectric ordering to PNRs. With an increase in electric field, the initially ordered short-range PNRs transition into long-range ferroelectric ordering, resulting in a continuous rise in *P*_max_. Thus, an increase in the external electric field led to a continuous increase in *W*_rec_ and *W*. The *E*_b_ values for 0.08CLMN, 0.10CLMN, 0.12CLMN, 0.14CLMN, and 0.16CLMN were determined to be 280, 340, 320, 300, and 290 kV cm^−1^, respectively. The *W*_rec_ value of the pure sample demonstrated an initial increase from 0.65 J cm^−3^ to a maximum of 3.40 J cm^−3^ at *x* = 0.10. However, further doping leads to a decline in *W*_rec_. The 0.10CLMN composition demonstrated the best energy storage performance, achieving a high value of *W*_rec_ (3.40 J cm^−3^) and *η* (81%). Therefore, the addition of CLMN can substantially enhance the *W* of BT-NBT-based ceramics.

Figure 6d presents a summary of key energy storage parameters at approximately 350 kV cm^−1^ for several lead-free dielectric ceramic materials, including bismuth ferrite (BiFeO_3_, BF)–BT-based [43,44,45], BT-based [23,30,39], potassium sodium niobate [(K, Na)NbO_3_, KNN]-based [46,47,48], and NBT-based [49,50,51,52,53] ceramics. High *ƞ* and *W*_rec_ of 0.10CLMN ceramic are achieved simultaneously, indicating it as a potential component for the application in energy storage devices. The energy storage properties of 0.10CLMN are comparable with most reported studies, with wide application prospects in dielectric capacitors. Table 1 presents a comparison of the energy storage characteristics of 0.10CLMN ceramic with those of other reported lead-free materials. The energy storage properties of 0.10CLMN specimen were found to be analogous to those of other reported Pb-free ceramics.

**Figure 7:**
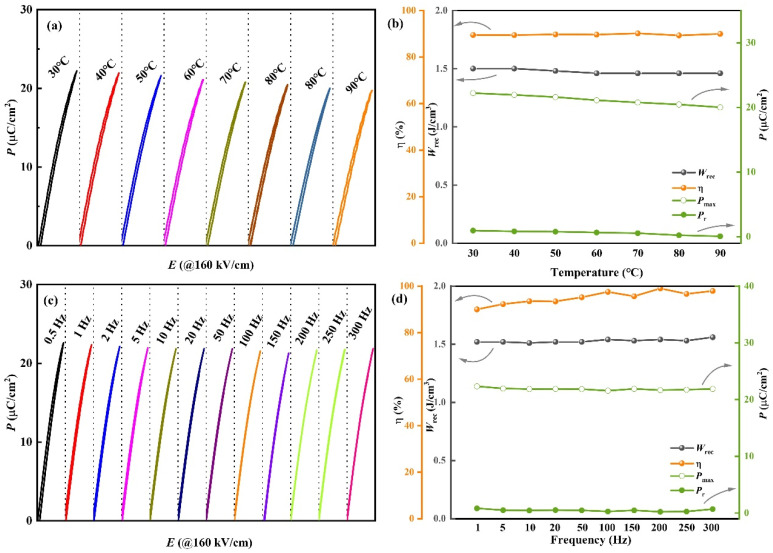
(**a**) Unipolar *P-E* curves (30–100 °C at 50 Hz at 160 kV/cm); (**c**) Unipolar *P-E* curves (50–300 Hz at room temperature at 160 kV/cm); (**b**,**d**) show the energy storage temperature, and frequency stability of the 0.10CLMN ceramic at 160 kV/cm.

While dielectric capacitors demonstrated high *W* and *η*, maintaining stable energy storage performance is essential for their reliable use in electrostatic capacitor applications. Moreover, the capacitors must also demonstrate consistent reliability. Since 0.10CLMN demonstrated high *η* and *W*_rec_, its *P*–*E* loops with an *E* of 160 kV cm^−1^ were selected for an in-depth analysis of energy storage performance under different temperature and frequency conditions (Figure 7a,c). The values of *P*_max_, *P*_r_, *W*_rec_, and *ƞ* of 0.10CLMN, calculated using the methods described previously [8] are presented in Figure 7b,d. Remarkably, the *P–E* loops of 0.10CLMN demonstrated a narrow shape. According to Figure 7b, the *P*_max_ and *P*_r_ demonstrated a slight decrease with rising temperature, whereas the values of *W*_rec_ and *ƞ* ranged from 1.46–1.50 J cm^−3^ and 89.28–90.17%, respectively. The observed fluctuations for these parameters (Δ*W*_rec_ < 2.67% and Δ*η* < 0.82%) are small within this temperature range. Thus, the energy storage performance of 0.10CLMN might possess high temperature stability. Figure 7d shows that the fluctuation of *P*_max_ and *P*_r_ at 0.5–300 Hz is relatively small. Thus, *W*_rec_ remains relatively stable around 1.50 J cm^−3^, with fluctuations not exceeding 2.63% across the frequency range of 0.5 to 300 Hz. The *η* of 0.10CLMN remained above 90% within this frequency range, demonstrating its potential for use across a broad spectrum of frequencies. These findings highlight the favorable energy storage performance of 0.10CLMN, characterized by its strong stability across both temperature and frequency variation.

## 4. Conclusions

By doping CLMN, a linear dielectric material, into BT–NBT–based ceramic materials, a substantial improvement in energy storage capability was achieved. The performed modification enhanced the insulation performance and altered the relaxation behavior, optimizing the overall energy storage properties of the ceramics. These modifications significantly enhanced the breakdown strength while reducing hysteresis loss, leading to a synergistic increase in both *W*_rec_ and *η*. *W*_rec_ of 3.40 J cm^−3^ and *ƞ* of 81% was achieved in the 0.10CLMN. The energy storage parameters demonstrated high thermal and frequency stability, with *W*_rec_ fluctuating by less than 2.67% and 2.63% over the 30–90 °C and 0.5–300 Hz range, respectively. Thus, 0.10CLMN is a promising material for pulsed-power applications and demonstrates a linear additive approach for developing ceramic materials with high energy storage performance.

## Figures and Tables

**Table 1:** Comparison of energy storage properties of 0.10CLMN ceramic with other ceramic capacitor systems.

Composition	*W*_rec_ (J cm^−3^)	*η*(%)	*E*_b_ (kV cm^−1^)	Refs.
BT–NBT–SBMT	7.12	90	720	[30]
BT–NBT–CZ	9.04	87.2	540	[19]
BT–BMH	3.38	87	240	[39]
BT–NBT–BZMASZ	3.74	82.2	273	[23]
BT–NBT–BMN	1.6	90.3	182	[24]
BF–BT–NNT	6.1	81.4	330	[54]
BF–BT–LA	5.71	80.19	270	[55]
BCT–BMZ	10.9	93	720	[56]
BNBSCTZZTN	7.3	90.6	530	[57]
KNN–STZ–BZTN	11.14	87.1	750	[46]
NBT–NN	8.04	85	630	[58]
BT–NBT–CLMN	3.40	81	340	This work

## Data Availability

The original contributions presented in this study are included in the article. Further inquiries can be directed to the corresponding author(s).

## References

[1] Wang T., Mallikarjuna K., Vattikuti S.V.P., Altaf M., Goud B.S., Koyyada G., Shim J. (2024). Synergistic enhancement of electrochemical storage using g- C_3_N_4_ modified MoO_2_/MoO_3_ nanostructure electrodes via thermal decomposition. J. Energy Storage.

[2] Chen L., Hu T.F., Shi X.M., Yu H.F., Zhang H., Wu J., Fu Z.Q., Qi H., Chen J. (2024). Near-zero energy consumption capacitors by controlling inhomogeneous polarization configuration. Adv. Mater..

[3] Cui C.H., Bai F., Yang Y.N., Hou Z.Q., Sun Z., Zhang T. (2024). Ion-exchange-induced phase transition enables an intrinsically air sable hydrogarnet electrolyte for solid-state lithium batteries. Adv. Sci..

[4] Duan J.H., Wei K., Du Q.B., Ma L.Z., Yu H.F., Qi H., Tan Y.C., Zhong G.K., Li H. (2024). High-entropy superparaelectrics with locally diverse ferroic distortion for high-capacitive energy storage. Nat. Commun..

[5] Yang B., Liu Y., Jiang R.J., Lan S., Liu S.Z., Zhou Z., Dou L., Zhang M., Huang H., Chen L.Q. (2025). Enhanced energy storage in antiferroelectrics via antipolar frustration. Nature.

[6] Zhang M., Lan S., Yang B.B., Pan H., Liu Y.Q., Zhang Q.H., Qi J.L., Chen D., Su H., Yi D. (2024). Ultrahigh energy storage in high-entropy ceramic capacitors with polymorphic relaxor phase. Science.

[7] Yang B., Zhang Y., Pan H., Si W., Zhang Q., Shen Z., Yu Y., Lan S., Meng F., Liu Y. (2022). High-entropy enhanced capacitive energy storage. Nat. Mater..

[8] Chen S., Wang T., Wang X.L., Li K., Zhu Q.F., Gong W.P., Liu G., Wang Q.Y., Xie S.X. (2025). Structural origin of enhanced storage energy performance and robust mechanical property in A-site disordered high-entropy ceramics. Rare Met..

[9] Chen L., Deng S., Liu H., Wu J., Qi H., Chen J. (2022). Giant energy-storage density with ultrahigh efficiency in lead-free relaxors via high-entropy design. Nat. Commun..

[10] Zhong W.T., Liu X.Y., Zheng X.T., Zheng P., Wang J.Q., Sheng L.S., Zheng L., Fan Q.L., Bai W.F., Zhang Y. (2025). Realizing exceptional energy storage performance in tungsten bronze-based ceramics via weakly coupled relaxor and grain boundary reinforcement designs. ACS Appl. Mater. Interfaces.

[11] Zhang L., Pu Y., Chen M., Peng X., Wang B., Shang J. (2023). Design strategies of perovskite energy-storage dielectrics for next-generation capacitors. J. Eur. Ceram. Soc..

[12] Tang T., Liu J.C., Liu D., Han Y., Luan R.D., Wang Q., Liu H., Zhang B.P., Zheng Q., Deng S.Q. (2025). Self-generated glass-ceramics-like structure boosts energy storage performance of AgNbO_3_-based MLCC. Adv. Funct. Mater..

[13] Tang T., Liu D., Wang L., Li J.Z., Zhang Z., Zhao L., Zhang B.P., Zhu L.F. (2025). Ultrahigh energy storage density and efficiency of antiferroelectric AgNbO_3_-based MLCCs via reducing the off-center cations displacement. Chem. Eng. J..

[14] Zhou J., Liu D.K., Chen R.X., Zhang K., Jin R.Q., Sun H.C., Feng Y.J., Wei X.Y., Xu Z., Xu R. (2024). Enhanced ultra-high efficiency in high-energy-density PbHfO_3_-based antiferroelectric ceramics through synergistic effect design. Chem. Eng. J..

[15] Wu L.W., Cai Z.M., Zhu C.Q., Feng P.Z., Li L.T., Wang X.H. (2020). Significantly enhanced dielectric breakdown strength of ferroelectric energy-storage ceramics via grain size uniformity control: Phase-field simulation and experimental realization. Appl. Phys. Lett..

[16] Wang W., Zhang L., Yang Y., Shi W., Huang Y., Alikin D.O., Shur V.Y., Lou Z., Zhang A., Wei X. (2023). Enhancing energy storage performance in Na_0.5_Bi_0.5_TiO_3_-based lead-free relaxor ferroelectric ceramics along a stepwise optimization route. J. Mater. Chem. A.

[17] Li D., Xu D., Zhao W., Avdeev M., Jing H., Guo Y., Zhou T., Liu W., Wang D., Zhou D. (2023). A high-temperature performing and near-zero energy loss lead-free ceramic capacitor. Energy Environ. Sci..

[18] Cao W., Lin R., Hou X., Li L., Li F., Bo D., Ge B., Song D., Zhang J., Cheng Z. (2023). Interfacial polarization restriction for ultrahigh energy storage density in lead free ceramics. Adv. Funct. Mater..

[19] Chen L., Li F., Gao B., Zhou C., Wu J., Deng S., Liu H., Qi H., Chen J. (2023). Excellent energy storage and mechanical performance in hetero-structure BaTiO_3_-based relaxors. Chem. Eng. J..

[20] Lv J., Li Q., Li Y., Tang M., Jin D., Yan Y., Fan B., Jin L., Liu G. (2021). Significantly improved energy storage performance of NBT-BT based ceramics through domain control and preparation optimization. Chem. Eng. J..

[21] Yuan Q.B., Li G., Yao F.Z., Cheng S.D., Wang Y.F., Ma R., Mi S.B., Gu M., Wang K., Li J.F. (2018). Simultaneously achieved temperature-insensitive high energy density and efficiency in domain engineered BaTiO_3_-Bi(Mg_0.5_Zr_0.5_)O_3_ lead-free relaxor ferroelectrics. Nano Energy.

[22] Zhu W., Shen Z.Y., Deng W., Li K., Luo W.Q., Song F.S., Zeng X.J., Wang Z.M., Li Y.M. (2024). A review: (Bi,Na)TiO_3_ (BNT)-based energy storage ceramics. J. Mater..

[23] Zhou S., Pu Y., Zhang X., Shi Y., Gao Z., Feng Y., Shen G., Wang X., Wang D. (2022). High energy density, temperature stable lead-free ceramics by introducing high entropy perovskite oxide. Chem. Eng. J..

[24] Qiu Y., Lin Y., Liu X.Y., Yang H.B. (2019). Bi(Mg_2/3_Nb_1/3_)O_3_ addition inducing high recoverable energy storage density in lead-free 0.65BaTiO_3_-0.35Bi_0.5_Na_0.5_TiO_3_ bulk ceramics. J. Alloys Compd..

[25] Dai Z., Xie J., Fan X., Ding X., Liu W., Zhou S., Ren X. (2020). Enhanced energy storage properties and stability of Sr(Sc_0.5_Nb_0.5_)O_3_ modified 0.65BaTiO_3_-0.35Bi_0.5_Na_0.5_TiO_3_ ceramics. Chem. Eng. J..

[26] Xie A., Fu J., Zuo R., Zhou C., Qiao Z., Li T., Zhang S. (2022). NaNbO_3_-CaTiO_3_ lead-free relaxor antiferroelectric ceramics featuring giant energy density, high energy efficiency and power density. Chem. Eng. J..

[27] Ye W.B., Zhu C.H., Xiao Y.M., Bai X.Z., Zheng P., Zhang J.J., Bai W.F., Fan Q.L., Zheng L., Zhang Y. (2023). Remarkable energy-storage performances and excellent stability in CaTiO_3_-doped BiFeO_3_-BaTiO_3_ relaxor ferroelectric ceramics. J. Eur. Ceram. Soc..

[28] Yang F., Pan Z., Ling Z., Hu D., Ding J., Li P., Liu J., Zhai J. (2021). Realizing high comprehensive energy storage performances of BNT-based ceramics for application in pulse power capacitors. J. Eur. Ceram. Soc..

[29] Wang T., Li Y., Zhang X., Zhang D., Gong W. (2023). Simultaneous excellent energy storage density and efficiency under applied low electric field for high entropy relaxor ferroelectric ceramics. Mater. Res. Bull..

[30] Chen X., Wang M., Pan Z., Li H., Zhao J., Tang L., Liu J., Li P., Xie H., Zhai J. (2023). Ultrahigh energy density and efficiency of BaTiO_3_-based ceramics via multiple design strategies. Chem. Eng. J..

[31] Li C., Liu J., Bai W., Wu S., Zheng P., Zhang J., Pan Z., Zhai J. (2022). Superior energy storage performance in (Bi_0.5_Na_0.5_)TiO_3_-based lead-free relaxor ferroelectrics for dielectric capacitor application via multiscale optimization design. J. Mater. Chem. A.

[32] Minakshi M., Samayamanthry A., Whale J., Aughterson R., Shinde P.A., Ariga K., Kumar Shrestha L. (2024). Phosphorous-containing activated carbon derived from natural honeydew peel powers aqueous supercapacitors. Chem. Asian J..

[33] Wickramaarachchi K., Sundaram M.M., Henry D.J., Gao X. (2021). Alginate biopolymer effect on the electrodeposition of manganese dioxide on electrodes for supercapacitors. ACS Appl. Energy Mater..

[34] Xie A., Zuo R., Qiao Z., Fu Z., Hu T., Fei L. (2021). NaNbO_3_-(Bi_0.5_Li_0.5_)TiO_3_ lead-free relaxor ferroelectric capacitors with superior energy-storage performances via multiple synergistic design. Adv. Energy Mater..

[35] Luo N.N., Han K., Cabral M.J., Liao X.Z., Zhang S.J., Liao C.Z., Zhang G.Z., Chen X.Y., Feng Q., Li J.F. (2020). Constructing phase boundary in AgNbO_3_ antiferroelectrics: Pathway simultaneously achieving high energy density and efficiency. Nat. Commun..

[36] Chen X.L., Li X., Sun J., Sun C.C., Shi J.P., Pang F.H., Zhou H.F. (2020). Achieving ultrahigh energy storage density and energy efficiency simultaneously in barium titanate based ceramics. Appl. Phys. A-Mater. Sci. Process..

[37] Yan F., Huang K.W., Jiang T., Zhou X.F., Shi Y.J., Ge G.L., Shen B., Zhai J.W. (2020). Significantly enhanced energy storage density and efficiency of BNT-based perovskite ceramics via A-site defect engineering. Energy Storage Mater..

[38] Zhao J., Pan Z., Tang L., Shen Y., Chen X., Li H., Li P., Zhang Y., Liu J., Zhai J. (2022). Greatly enhanced discharged energy density and efficiency of BiFeO_3_-Based ceramics by regulating insulation performance. Mater. Today Phys..

[39] Liu Z.G., Li M.D., Tang Z.H., Tang X.-G. (2021). Enhanced energy storage density and efficiency in lead-free Bi(Mg_1/2_Hf_1/2_)O_3_-modified BaTiO_3_ ceramics. Chem. Eng. J..

[40] Guan Z.N., Yan Y., Ma J., Pan T., Li X., Guo S., Zhang J., Wang J., Wang Y. (2022). Significantly enhanced energy storage performance of lead-free BiFeO_3_-based ceramics via synergic optimization strategy. ACS Appl Mater Interfaces.

[41] Deng X.Z., Zhang L.Y., Geng X.Y., Zhang J., Sun L., Wang R.-X., Gu Z.-B., Zhang S.-T. (2017). Crystal structure, impedance, and multiferroic property of SrZrO_3_ and MnO_2_ modified 0.725BiFeO_3_-0.275BaTiO_3_ ceramics. Ceram. Int..

[42] Weng N., Zhang J., Wang Z.Y., Wang H., Wang L., Wang J., Wang Y.J. (2024). Moderate electric field driven ultrahigh energy density in BiFeO_3_-BaTiO_3_-based ceramics with improved relaxor behavior and breakdown strength. Chem. Eng. J..

[43] Zhao J., Li H., Du Y., Chen X., Qin H., Wang J., Yan T., Yu S., Hu Y., Wang D. (2024). Superior energy storage performance of BiFeO_3_-BaTiO_3_-CaHfO_3_ lead-free ceramics. J. Mater. Chem. A.

[44] Montecillo R., Chien R.R., Chen C.S., Wu P.H., Tu C.S., Feng K.C. (2024). Ultrahigh energy storage in multilayer BiFeO_3_-BaTiO_3_-NaTaO_3_ relaxor ferroelectric ceramics. J. Mater. Chem. A.

[45] Zhao J., Hu T., Fu Z., Pan Z., Tang L., Chen X., Li H., Hu J., Lv L., Zhou Z. (2023). Delayed polarization saturation induced superior energy storage capability of BiFeO_3_-based ceramics via introduction of non-isovalent ions. Small.

[46] Huan Y., Wu L.Z., Xu L.Y., Li P., Wei T. (2025). Superior energy-storage density and ultrahigh efficiency in KNN-based ferroelectric ceramics via high-entropy design. J. Mater..

[47] Zha J.L., Yang Y.L., Liu J.X., Lu X.M., Hu X.L., Yan S., Wu Z.J., Zhou M., Huang F.Z., Ying X.N. (2024). High energy storage performance of KNN-based relaxor ferroelectrics in multiphase-coexisted superparaelectric state. J. Appl. Phys..

[48] Sun Z., Zhao S., Wang T., Jing H., Guo Q., Gao R., Diwu L., Du K., Hu Y., Pu Y. (2024). Achieving high overall energy storage performance of KNN-based transparent ceramics by ingenious multiscale designing. J. Mater. Chem. A.

[49] Mao P., Guo Y., Lu G., Yan Q., Kang R., Wang T., Xie B., Liu Z., Zhang L. (2023). Synergistic effect of multi-phase and multi-domain structures induced high energy storage performances under low electric fields in Na_0.5_Bi_0.5_TiO_3_-based lead-free ceramics. Chem. Eng. J..

[50] Zhu X., Gao Y., Shi P., Kang R., Kang F., Qiao W., Zhao J., Wang Z., Yuan Y., Lou X. (2022). Ultrahigh energy storage density in (Bi_0.5_Na_0.5_)_0.65_Sr_0.35_TiO_3_-based lead-free relaxor ceramics with excellent temperature stability. Nano Energy.

[51] Liu X., Hou Y., Song B., Cheng H., Fu Y., Zheng M., Zhu M. (2022). Lead-free multilayer ceramic capacitors with ultra-wide temperature dielectric stability based on multifaceted modification. J. Eur. Ceram. Soc..

[52] Zhao X., Zhang L., Fan Z., Huang Y., Hu Y., Shen M., Wang Z., He Y., Wang D., Zhang Q. (2025). Excellent high-temperature dielectric energy storage performance in bilayer nanocomposites with high-entropy ferroelectric oxide fillers. Nat. Commun..

[53] Fan Z., Dai J., Huang Y., Xie H., Jiao Y., Yue W., Huang F., Deng Y., Wang D., Zhang Q. (2025). Superior energy storage capacity of polymer-based bilayer composites by introducing 2D ferroelectric micro-sheets. Nat. Commun..

[54] Dang S., Peng Z., Zhang X., Wang Y., Chai Q., Wu D., Liang P., Wei L., Chao X., Yang Z. (2025). Enhanced energy storage performance of BiFeO_3_-BaTiO_3_ based ceramics under moderate electric fields via multiple synergistic design. Chem. Eng. J..

[55] Zhang J.B., Pu Y.P., Hao Y.X., Yang Y.L., Zhang L., Wang B., Pan Q. (2025). Realizing excellent energy-storage performance under low electric fields in lead-free BiFeO_3_-BaTiO_3_-based ceramics with ultrahigh polarization difference. J. Energy Storage.

[56] Kong X., Yang L.T., Meng F.Q., Zhang T., Zhang H.J., Lin Y.H., Huang H.B., Zhang S.J., Guo J.M., Nan C.W. (2025). High-entropy engineered BaTiO_3_-based ceramic capacitors with greatly enhanced high-temperature energy storage performance. Nat. Commun..

[57] Zheng B., Yuan Q., Lin Y., Li D., Yang H., Hong Z., Ma Y., Ma Y., Guo J., Wang J. (2025). High entropy-driven large capacitive energy storage in BaTiO_3_-based multilayer ceramic capacitors. Adv. Energy Mater..

[58] Jiang Y., Liu J.M., Zhang W.C., Cheng X., Hui K.Z., Zhen Y.C., Hao Y.N., Bi K., Guo L.M., Zhao P.Y. (2025). Comprehensively improved energy storage and DC-bias properties in Bi_0.5_Na_0.5_TiO_3_-NaNbO_3_ based relaxor antiferroelectric. J. Mater..

